# Quality of consumer-oriented websites containing information about the second trimester ultrasound examination during pregnancy

**DOI:** 10.1186/s12884-020-02897-w

**Published:** 2020-04-22

**Authors:** Susanne Georgsson, Tommy Carlsson

**Affiliations:** 1 The Swedish Red Cross University College, Box 1059, SE-14121 Huddinge, Sweden; 2 Karolinska Institutet, Department of Clinical science, Intervention and technology, SE-17177 Stockholm, Sweden; 3 Department of Women’s and Children’s Health, MTC-huset, Dag Hammarskjölds väg 14B, 1 tr, Uppsala University, SE-75237 Uppsala, Sweden

**Keywords:** Consumer health information, Pregnancy, Second pregnancy trimester, Prenatal care, Ultrasonography, World wide web

## Abstract

**Background:**

Providing information about prenatal tests is a clinical challenge and the public frequently accesses the Web to read pregnancy-related information. The overarching aim of this study was to investigate the quality of consumer-oriented websites addressing obstetric ultrasound examination in the second trimester of pregnancy.

**Methods:**

Swedish websites were identified with Google, using 20 search strings and screening 400 hits (*n* = 71 included websites). Reliability and information about the examination were assessed with the DISCERN instrument, completeness was assessed according to national guidelines, and readability analyzed with the Readability Index. Popularity was determined with the ALEXA tool and search rank was determined according to Google hit lists.

**Results:**

The mean total DISCERN score was 29.7/80 (SD 11.4), with > 50% having low quality for 15 of the 16 questions. The mean completeness score was 6.8/24 (SD 4.5). The Readability Index ranged between 22 and 63, with a mean of 42.7 (SD 6.8), indicating difficult readability. Weak and non-significant correlations were observed between ALEXA/search rank and the investigated quality variables, except for search rank and reliability.

**Conclusions:**

The quality of consumer-oriented websites addressing the second trimester ultrasound examination is low. Health professionals need to discuss this with expectant parents considering undergoing the examination. There is a need for efforts that aim to improve the poor quality of online sources in the field of prenatal examinations.

## Background

When second trimester obstetric ultrasound examinations are offered routinely, most expectant parents accept the offer to undergo it and find the decision easy to reach [1]. Expectant parents regard the examination as a positive event in which they expect to be presented with a visual confirmation of the pregnancy and to be reassured about the health of their expected child [2]. However, there is an identified discrepancy between expectant parents’ expectations and the medical purposes of the examination, calling attention to the importance of pre-ultrasound information [3]. Research shows that while most expectant parents are satisfied with the pre-ultrasound information, they still experience a lack of information about certain aspects [4]. Moreover, those who receive news about a medical condition feel ill prepared and experience psychological distress [5]. Various methods to offer information to expectant parents have been tested, but no standard that improves knowledge more than usual care has yet emerged [6]. Providing information about prenatal tests is a clinical challenge for health professionals who work in maternity care. These health professionals express a lack of training about prenatal diagnostics [7] and often devote insufficient time to inform expectant parents about the examinations [8], indicating that they are unable to provide sufficient information about the different alternatives.

Today, the Web is a highly popular source of information about reproductive health, including pregnancy-related information [9]. Studies show that most turn to the Web first when having a health-related query and that very few decide to talk directly to a health care provider instead [10], indicating that the Web now dominates as a first-line source of information. The Web has the potential to serve as a source of tailored and multimodal information that could aid in making health-related decisions through the dissemination of information and possibilities to promote interaction between health professionals and service users [11]. More specifically, high-quality and accessible web-based information could potentially help expectant parents decide whether or not to undergo the second trimester ultrasound examination [12]. However, the unregulated structure of the Web, including the lack of peer review processes or other regulatory activities, entails a risk that consumers can encounter information of low quality [13–15]. The quality of web-based sources has been questioned repeatedly over the course of several years across different health contexts [13, 16–18]. The multidimensional concept of quality of web-based information encompasses many aspects [13, 19], with the most commonly assessed in research being accuracy, completeness, content, readability, design, disclosures, and use of references [13]. Consumers who turn to the Web need to have the skills to identify not only relevant and trustworthy sources, but also to read and understand the information encountered, so that they can reach informed decisions [12]. While some studies report quality deficits for websites about non-invasive prenatal testing [20–22], knowledge is limited concerning the quality of web-based information about the second trimester ultrasound examination. Investigating the quality of consumer-oriented sources may result in valuable insights as to how to discuss use of the Web when counseling expectant parents. The overarching aim of this study was to investigate the quality of consumer-oriented websites addressing obstetric ultrasound examination in the second trimester of pregnancy. Specifically, the purpose was to assess the reliability, quality of information about the examination, completeness and readability of these websites. Further, we set out to investigate potential associations between the investigated quality criteria and website popularity or search rank.

## Methods

### Study context

This study concerns Swedish websites. In Sweden, obstetric ultrasound examinations are offered to all pregnant women in the second trimester of pregnancy. The examination is used to calculate gestational age, localize the placenta, determine number of fetuses, screen for fetal anomalies, and estimate volume of amniotic fluid. Midwives in maternity care services provide information about the examination and schedule an examination for those who want to undergo the procedure. Specialist midwives or obstetricians perform the examination. There is a very high accessibility to the Internet and almost all Swedes use the Web as a source of information [23].

### Data collection

The methods in this study adhere to current guidelines for systematic evaluations of websites as described in the literature [24]. We designed the searches to resemble search patterns among the public, based on the results of previous research. This involves using various types of search strings, limiting the data collection to the first web page presented when accessing the link in the hit list, and screening the first ten links of the hit list before moving on to a new search [25–27]. Swedish websites about the obstetric ultrasound examination in the second trimester of pregnancy were identified through searches in Google, the most used search engine on the Web [23]. In total, we designed 20 search strings consisting of both medical terminology as well as generic terms used by the public (Table 1). To check for common search terms used by the public in Sweden, Google Trends was used. We explored search terms in the categories “*obstetric ultrasonography*” and “*ultrasound midwives*”, as well as related search terms for each of the 20 chosen search strings. This did not reveal any alternative search terms and validated our chosen search strings. The searches were performed in January 2019. The hit list in the search engine ranged from 35,900 to 32,100,000.

**Table 1:** Search strings, total hits and included hits

Search string in Swedish	Search string translated in English	Total hits	Included hits	
			Unique	Duplicate
*Ultraljud gravid*	*Ultrasound pregnant*	1,150,000	13	1
*Ultraljud graviditet*	*Ultrasound pregnancy*	744,000	2	10
*Rutinultraljud*	*Routine ultrasound*	85,100	9	1
*Ultraljud*	*Ultrasound*	3,480,000	6	3
*Ultraljud på gravida*	*Ultrasound of pregnant*	1,150,000	1	11
*Ultraljud på foster*	*Ultrasound of fetuses*	189,000	1	11
*Ultraljud bebis*	*Ultrasound baby*	514,000	2	9
*Ultraljud foster avvikelse*	*Ultrasound fetal anomaly*	35,900	2	4
*Ultraljud missbildning*	*Ultrasound congenital malformation*	48,500	1	4
*Ultraljud andra trimestern*	*Ultrasound second trimester*	80,400	0	4
*Foster undersökning*	*Fetus examination*	393,000	2	7
*Undersökning gravid*	*Examination pregnant*	1,830,000	8	4
*Vad kan ses på ultraljudet under graviditeten*	*What can be seen on the ultrasound during pregnancy*	349,000	4	10
*Hur fungerar ultraljud vid graviditet*	*How does ultrasound in pregnancy work*	1,300,000	2	13
*Ultraljud hos barnmorskan*	*Ultrasound at the midwife*	282,000	7	5
*RUL*	*RUL [Common abbreviation for routine ultrasound]*	32,100,000	0	3
*Vad är RUL*	*What is RUL [common abbreviation for routine ultrasound]*	290,000	3	7
*Fosterdiagnostik*	*Fetal diagnostics*	123,000	7	3
*Ska jag göra rutinultraljudet*	*Should I undergo the routine ultrasound*	62,100	0	10
*Ultraljud vecka*	*Ultrasound week*	896,000	1	7
TOTAL FOR ALL SEARCH STRINGS			71	127

**Fig. 1:**
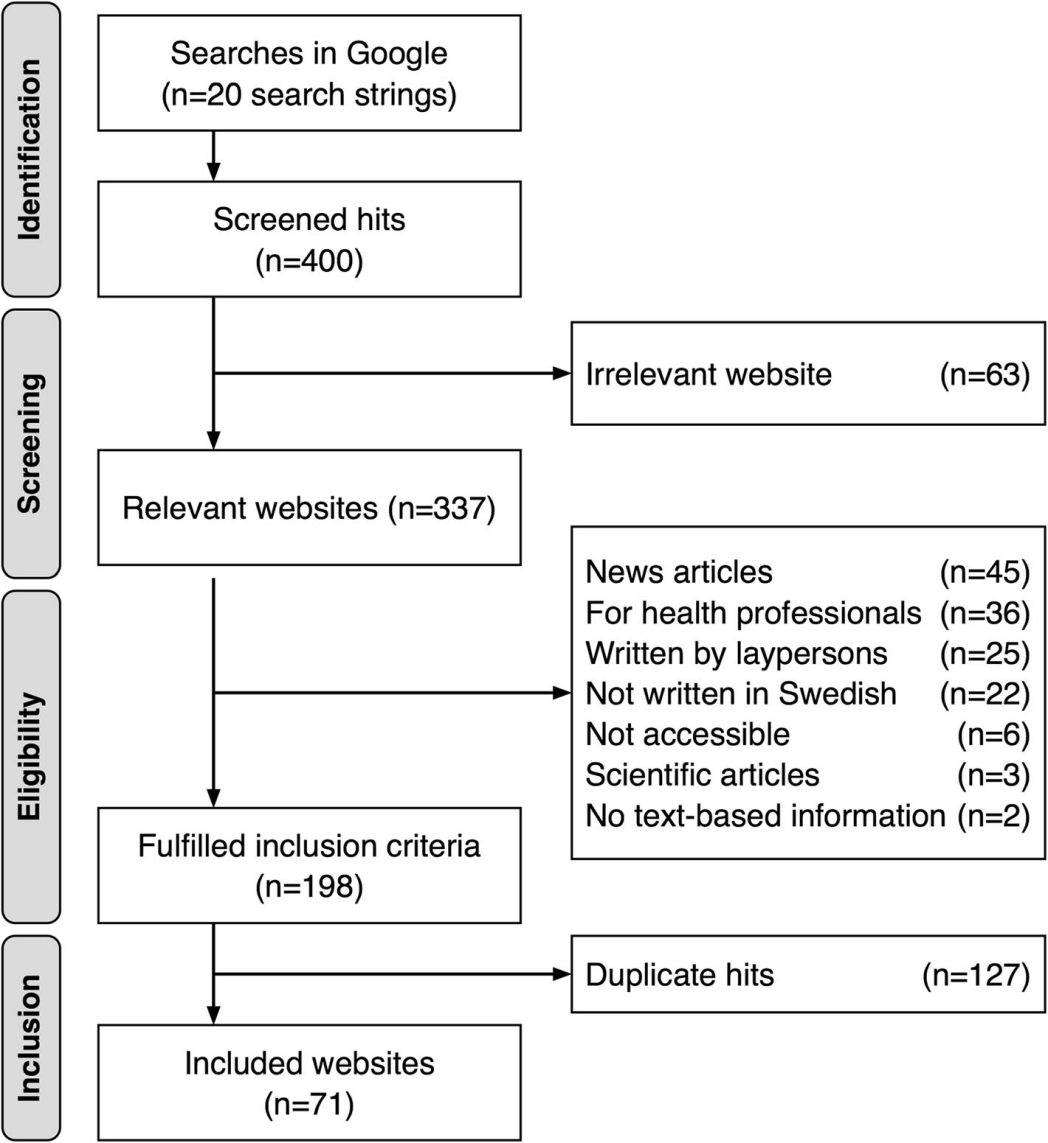
Search process for consumer-oriented websites about second trimester ultrasound examination

The first 20 hits of each search string were screened for inclusion, resulting in 400 hits screened in total. To be included, the website needed to: (1) contain information about the obstetric ultrasound examination in the second trimester of pregnancy, (2) include text-based information in Swedish, (3) provide information developed for consumers, i.e. expectant parents who search for information about the examination, and (4) be publicly accessible without the need for a password. In total, 63 (16%) hits led to irrelevant information, i.e. websites that did not contain any information about the obstetric ultrasound examination in the second trimester of pregnancy. Of the remaining relevant websites (*n* = 337), 139 (41%) hits were excluded because they were news articles, included information for health professionals, were written by laypersons to communicate with peers, were not written in Swedish, were inaccessible, were scientific articles and did not contain any text-based information. After correcting for duplicate hits (*n* = 127), 71 websites were included (Fig. 1). The final sample originated from independent information websites (*n* = 29), healthcare system (*n* = 27), government (*n* = 9), pharmaceutical companies/pharmacies (*n* = 3), charities/private organizations (n = 2), and a museum (*n* = 1). All included websites were saved with Webcite, an online archiving system for web-based sources.

### Data analysis

The analysis was guided by current recommendations for systematic evaluation of web-based information [24], including reliability, quality of information about the examination, comprehensiveness, and readability. The last author, a specialist nurse-midwife and researcher, assessed the included websites. Statistical analyses were performed with RStudio (version 1.0.143).

#### Reliability and quality of information about the examination

The DISCERN instrument, which is a valid and reliable tool [28], was used to assess the reliability and quality of information about the examination. DISCERN is used extensively in research and is recommended in the literature as a measure of quality in web-based sources [24]. In total, the instrument includes three subscales: reliability (eight questions), information about the examination (seven questions), and overall quality (one question). Each of the 16 questions was rated on a scale from 1 (no/low quality) to 5 (yes/high quality), resulting in a total score ranging from 16 to 80.

#### Completeness

An instrument was developed to assess completeness, inspired by national guidelines for information about prenatal screening (Table 2) [29]. In total, the instrument included 24 dichotomous questions. Each question rated “yes” received a score of 1, resulting in a total score ranging from 0 to 24.

**Table 2:** Instrument for assessment of completeness (criteria answered with yes or no)

Quality criteria	
Authors have formal education in health care services^a^	
Material developed in collaboration with patient representatives or patient associations	
Contains information that the examination is voluntary	
Contains information about the possibility of deliberation before examination	
Contains information about the following medical purposes of the examination:	
Calculate gestational age	
Determine number of fetuses	
Screen for fetal anomalies	
Localize placenta	
Estimate volume of amniotic fluid	
Contains information about other prenatal diagnostic tests:	
Non-invasive tests (e.g. analysis of cell-free DNA, combined first-trimester screening)	
Invasive tests (e.g. amniocentesis)	
Contains information that several prenatal examinations can be combined	
Contains information about how the examination works	
Contains information about indications for invasive prenatal tests	
Contains information about ethical aspects	
The information can be tailored depending on the user’s preferences and needs	
Contains information about which types of fetal anomalies that can be discovered	
Contains information about risks with the examination	
Contains information about risks with invasive prenatal tests	
Contains information about birth defects^b^	
Refers to additional information about birth defects	
Contains information about termination of pregnancy^c^	
Refers to additional information about termination of pregnancy	
Contains information about psychosocial support before and/or after prenatal examination^d^	

^a^ e.g. midwife or physician; ^b^ e.g. quality of life for those living with a birth defect, medical, social and psychological consequences when living with a birth defect, support from society when living with a birth defect, and how to come in contact with associations for children with birth defects that provide peer support; ^c^ e.g. legal possibilities and which methods used for induced abortion; ^d^ e.g. how to come in contact with a social worker or psychologist

#### Readability

The readability of the text-based material was analyzed with the automated calculation Readability Index [Läsbarhetsindex] (LIX), developed to compute readability of Swedish texts. Scores range from < 25 (easiest) to > 60 (most difficult). Scores > 40 indicate that the text is too difficult for persons with average literacy levels to understand fully [30].

#### Popularity and search rank

The ALEXA tool [31] was used to explore domain popularity. This tool produces a rank estimate of popularity in a specific country, such as Sweden. The rank calculation is based on unique visitors per day and page views on the site over the past month. The domain with the highest combination of average visitors per day and page views over the past month is ranked number one, meaning that high popularity results in a low rank score. Search rank was determined by the highest number of each included website, as presented in the hit list in Google. Spearman’s rank correlation test was used to determine correlations between popularity/highest search rank and the investigated quality criteria. *P*-values <.05 were considered statistically significant.

## Results

### Reliability and information about the examination

**Fig. 2:**
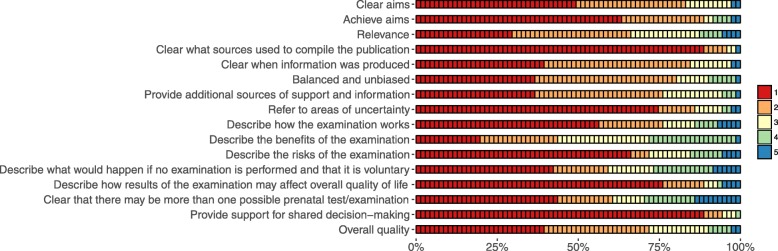
DISCERN quality scores for the included websites (*n* = 71), ranging from 1 (no/low quality) to 5 (yes/high quality)

The mean total DISCERN score was 29.7 (SD 11.4), illustrating poor quality (Table 3). In total, > 50% of the included websites had scores illustrating low quality (1–2) for 15 of the 16 questions (Fig. 2). The questions with the highest proportion of websites with low quality scores (1–2) were: “*Is it clear what sources of information were used to compile the publication?*” (*n* = 68, 96%), “*Does it provide support for shared decision-making?*” (*n* = 67, 94%), and “*Does it describe how results of the examination may affect overall quality of life?*” (*n* = 63, 89%). In contrast, the questions with the highest proportion of websites with high quality scores (4–5) were: “*Is it clear that there may be more than one possible prenatal test/examination?*” (*n* = 21, 30%), “*Does it describe the benefits of the examination?*” (*n* = 20, 28%), and “*Does it describe what would happen if no examination is performed and that it is voluntary?*” (*n* = 19, 27%). Approximately three quarters of the included websites were rated low quality (1–2) for subscale 3, illustrating low *overall quality* with serious or extensive shortcomings (*n* = 51, 72%).

**Table 3:** Means, standard deviations and ranges for the investigated quality measures (minimum to maximum achievable scores in square brackets)

Quality measure	M (SD)	Range
Reliability and information about the examination (DISCERN)		
Reliability (Subscale 1) [8–40]	13.9 (4.8)	8–32
Information about the examination (Subscale 2) [7–35]	13.8 (6.0)	7–33
Overall quality (Subscale 3) [1–5]	2.0 (1.1)	1–5
Total score [16–80]	29.7 (11.4)	16–68
Completeness (Based on national guidelines) [0–24]	6.8 (4.5)	0–20
Readability (Readability Index: LIX) [> 40: too difficult for persons with average literacy levels to fully understand]	42.7 (6.8)	22–63

### Completeness

**Fig. 3:**
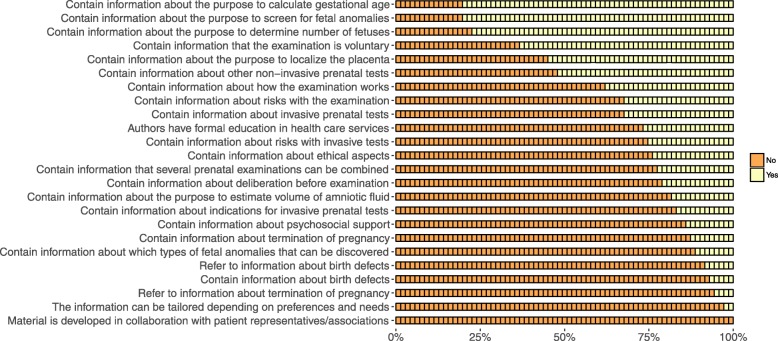
Completeness quality criteria for the included websites (*n* = 71)

The mean completeness score was 6.8 (SD 4.5) (Table 3). For 18 of the total 24 completeness quality criteria, > 50% of the websites did not adhere to the criteria (Fig. 3). The criteria with the lowest proportion of websites were *contains information about termination of pregnancy* (*n* = 0, 0%), *the information can be tailored depending on preferences and needs* (*n* = 2, 3%), and *refers to information about termination of pregnancy* (*n* = 4, 6%). In contrast, the criteria with the highest proportion of websites were: *contains information about the purpose to calculate gestational age* (*n* = 57, 80%), *contains information about the purpose to screen for fetal anomalies* (n = 57, 80%), and *contains information about the purpose to determine number of fetuses* (*n* = 55, 77%).

### Readability

The mean LIX was 42.7 (SD 6.8) (Table 3). The majority of the websites had LIX > 39 (*n* = 51, 72%), indicating that the texts had readability levels too difficult for average persons to understand fully (Table 4).

**Table 4:** Readability Index [Läsbarhetsindex: LIX] for the included websites (n = 71)

LIX score	What the score represent	n (%)
< 25	Easy-to-read, children’s books	2 (3%)
25–29	Easy level, fiction	1 (1%)
30–39	Moderate level, newspapers	17 (24%)
40–49	Difficult level, official texts	43 (61%)
50–60	Very difficult level, bureaucratic texts	7 (10%)
> 60	Highest difficulty level, dissertations	1 (1%)

### Popularity and search rank

**Fig. 4:**
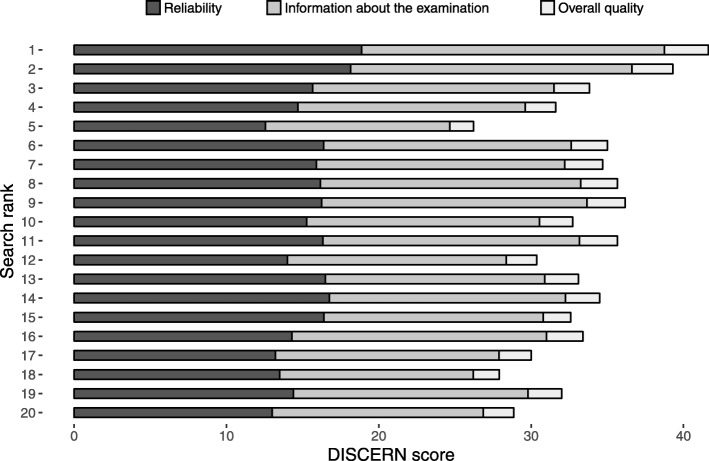
Mean DISCERN score for each search rank

**Fig. 5:**
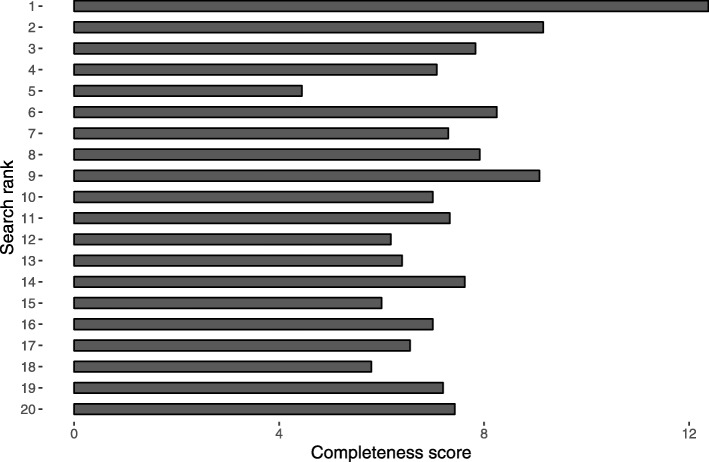
Mean completeness score for each search rank

**Fig. 6:**
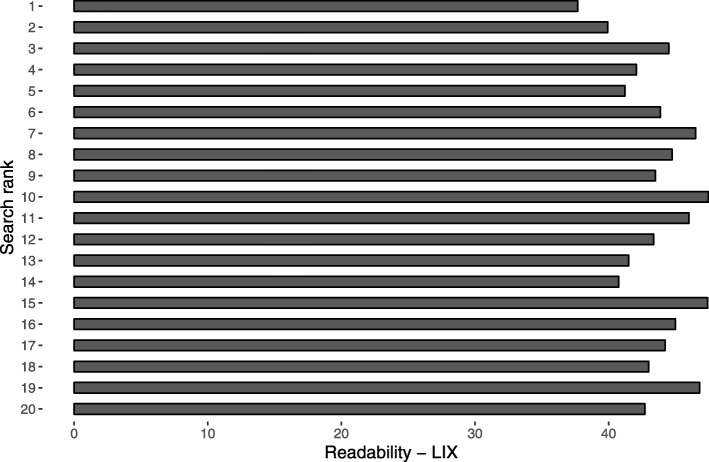
Mean readability index (LIX) for each search rank

The mean ALEXA rank score was 99.9 (range 1.4–862.0), indicating a high variability with regard to popularity. Weak and non-significant correlations were observed between ALEXA rank score and investigated quality variables (Table 5). There were weak and non-significant correlations between Google search rank and the investigated quality variables, except for reliability (*r*_*s*_ = − 0.28, *P* = 0.02). Please see the figures for a presentation of the mean DISCERN scores (Fig. 4), mean completeness score (Fig. 5), and mean LIX (Fig. 6), for each rank in the 20 searches.

**Table 5:** Spearman’s rank correlation coefficients and P-values with regard to the investigated quality variables and rank scores (ALEXA rank score and Google search rank)

Quality variable	ALEXA rank score		Google search rank	
	r_**s**_	P	r_**s**_	P
DISCERN subscale 1 (reliability)	0.23	0.09	−0.28	0.02*
DISCERN subscale 2 (quality of information)	0.13	0.33	−0.17	0.16
DISCERN subscale 3 (overall quality)	0.18	0.18	−0.21	0.08
Total DISCERN score	0.18	0.19	−0.22	0.06
Completeness score	0.22	0.12	−0.16	0.17
Readability Index (LIX)	−0.16	0.26	−0.02	0.86

*P < 0.05

## Discussion

The overarching aim of this study was to investigate the quality of websites addressing obstetric ultrasound examination in the second trimester of pregnancy. The reliability and quality of information about the examination was low, the completeness according to national guidelines was insufficient, and readability was difficult. There were no association between popularity or search rank and the investigated quality criteria.

According to previous studies, health professionals report insufficient training about prenatal tests [7], devote little time or effort to inform about prenatal tests [3, 8] and do not provide expectant parents with enough information about the option to terminate the pregnancy when a fetal anomaly is discovered [8]. Indeed, research illustrates that those who receive a prenatal diagnosis of fetal anomaly experience an overwhelming amount of information following the diagnosis [32, 33], and at the same time a lack of information about induced abortion [33, 34]. The websites had difficult readability, which further complicates the situation concerning informational uptake. Limited health literacy is prevalent and there is a need for improvement with regard to the readability levels of health-related information [35]. Difficult readability has been reported for web-based information in various other fields, including pregnancy-related topics [36]. While the overall quality of websites may be low, current algorithms in search engines aim to produce hit lists with links to high-quality websites as the first hits. Indeed, studies on search strategies among the public show that most rely on the first links in the hit list and the most popular websites [25, 26]. Thus, high-ranking websites and popular domains will have the highest impact and spread among information consumers [37]. However, our results indicate no association between popularity/search rank and quality, illustrating that parents who use the most common methods for web-based searches encounter websites of low quality. Combined, the results indicate that there is a need for studies that aim to help expectant parents search for and identify web-based information. Health professionals need to be mindful of the risk of low-quality websites when being consulted by expectant parents and raise this topic for discussion. There is a need for considerable and systematic undertakings that aim to improve the quality of web-based materials about prenatal tests.

There are methodological limitations that need to be considered when interpreting the findings. The sample consisted of Swedish websites addressing second trimester obstetric ultrasound examinations. Worldwide, there are regional differences regarding maternity care services and offers of prenatal tests. This needs to be considered when interpreting the results of this study, as the generalizability may be limited. We aimed to replicate the search patterns of expectant parents. Thus, we based our method on previous reports of how the public use the Web to find health-related information [25, 26]. In total, 400 hits generated by 20 different search strings in Google were screened, which is by far the most used search engine on the Web [23]. We cannot dismiss the possibility that expectant parents who search for websites with information about the ultrasound examination would encounter other websites than those identified in this study. It is also not possible to know how often expectant parents access the included websites. That the searches resulted in duplicate hits, meaning that several search terms led to the same websites, indicates saturation with regard to screened hits and that it is likely that expectant parents who search for Swedish information about the ultrasound examination are presented with a link to the included websites in their hit lists. This study investigated quality of consumer-oriented websites because we wanted the results to portray information developed with the purpose of providing information for expectant parents. Web-based information written for health professionals and those written by laypersons with the purpose of communicating with peers (e.g. blogs or discussion boards) were excluded. Expectant parents may decide to access these other types of websites to read about the ultrasound examination, which is not reflected in the results of this study. We acknowledge that the results may have limited generalizability due to the identification and inclusion of websites, and encourage more studies in this field so that a comprehensive understanding of the quality of these sources may be achieved.

The quality of the included websites was assessed with a focus on reliability, quality of information about the examination, completeness, and readability. That we assessed four aspects of website quality strengthens the results and is in line with current recommendations in the literature [24]. The DISCERN instrument was used to assess the reliability and the quality of information about the examination, which is a reliable and valid tool developed by experts and patient representatives. The last author, a researcher specialist nurse-midwife with previous experience of website assessments with standardized instruments, assessed the included websites. It has been shown previously that health professionals and laypersons assess website quality similarly when using the DISCERN instrument [38]. Nevertheless, we acknowledge that possibility that expectant parents may assess website quality differently and we would like to encourage more studies within this field of research.

## Conclusion

The quality of consumer-oriented websites addressing second trimester ultrasound examination is low regarding reliability, information about the examination, completeness and readability. Expectant parents who search for supplemental web-based information are at risk of encountering unreliable and incomplete information that has difficult readability and does not contain sufficient information about the examination. Midwives and physicians who work in maternity care need to discuss the risk of encountering low-quality web-based information about the second trimester ultrasound when consulting expectant parents who consider undergoing the examination. The high variability regarding completeness, and the fact that many topics highlighted in national guidelines were missing in many of the included websites, indicate that websites collectively do not sufficiently facilitate informed decisions whether or not to undergo the examination. Expectant parents are at risk of encountering information with difficult readability levels, which needs to be considered when consulting expectant parents, particularly those with low health literacy levels. There is a need for overarching clinical efforts and research that aim to guide expectant parents to high-quality online sources and improve the poor quality of web-based sources about prenatal examinations.

## Data Availability

The datasets used and/or analysed during the current study are available from the corresponding author on reasonable request.

## Abbreviation

LIX: Readability Index [Läsbarhetsindex]
